# Estimated adherence to the Dapivirine Vaginal Ring and its associated factors among African women: A systematic review and meta-analysis

**DOI:** 10.1371/journal.pgph.0006422

**Published:** 2026-05-18

**Authors:** Roseline Dzekem Dine, Giulia M. Muraca, Behnam Sadeghirad, Rachel Couban, Agatha Nyambi, Jéssyca Silva, Lawrence Mbuagbaw

**Affiliations:** 1 Department of Health Research Methods, Evidence and Impact, McMaster University, Hamilton, Ontario, Canada; 2 Department of Social Sciences and Community Engagement, Rinda Ubuzima, Kigali, Rwanda; 3 Department of Obstetrics and Gynecology, McMaster University, Hamilton, Ontario, Canada; 4 Clinical Epidemiology Unit, Department of Medicine, Solna, Karolinska Institutet, Eugeniahemmet, Stockholm, Sweden; 5 Department of Anesthesia, McMaster University, Hamilton, Ontario, Canada; 6 Michael G. DeGroote Institute for Pain Research and Care, McMaster University, Hamilton, Canada; 7 Department of Pediatrics, McMaster University, Hamilton, Ontario, Canada; 8 Biostatistics Unit, Father Sean O’Sullivan Research Centre, St Joseph’s Healthcare, Hamilton, Ontario, Canada; 9 Centre for Development of Best Practices in Health (CDBPH), Yaoundé Central Hospital, Yaoundé, Cameroon; 10 Division of Epidemiology and Biostatistics, Department of Global Health, Stellenbosch University, Cape Town, South Africa; London School of Hygiene and Tropical Medicine, UNITED KINGDOM OF GREAT BRITAIN AND NORTHERN IRELAND

## Abstract

The Dapivirine Vaginal Ring (DVR), a silicone circular ring, continuously distributes Dapivirine into the vagina to prevent HIV infection. We summarized evidence on adherence to DVR for HIV-1 prevention among African women 16–45 years old and described the factors associated with adherence. We searched different databased from inception to December 2024. We included observational studies and randomized trials. Our primary outcome was adherence to DVR, explored as women adhering, mean adherence, and visit/ring adherence. Pairs of reviewers independently screened for eligible studies and extracted relevant data. We performed a random-effects meta-analysis of proportions and means for DVR adherence. The certainty of the evidence was assessed using the GRADE approach. Our search retrieved 217 records, of which 15 articles published between 2016 and 2024 were found (seven unique studies). Across the seven included studies, 2,424 women using DVR were reported in four studies. Across the included studies, adherence to the vaginal ring was measured as proportion with good adherence (4 studies), mean adherence (1 study), number of follow-ups with good adherence (1 study); and 1 study reported both mean adherence and number of returned rings deemed adherent. Adherence to the DVR among women was generally good, with a pooled mean estimate of 84% (95% CI: 75%–91%), although the certainty of evidence was very low. The proportion of women who adhered was 76% (95% CI: 63%–87%). Appropriate use of rings was 74% (95% CI 74–75%) and visits with adherence reported was 89% (95% CI 89–90%). Factors influencing adherence were cross-cutting across multiple domains. The evidence suggests generally good adherence to the DVR, which may enhance its effectiveness in reducing HIV infections in real-world settings; however, the certainty of this evidence is low. We encourage further implementation research, particularly individual patient data meta-analyses, to assess adherence more accurately.

## Background

In 2023, the global prevalence of HIV was estimated at 39.9 million, with approximately 1.3 million new infections. Girls and women represented 53% of prevalent cases and 44% of all new infections [1]. Pre-Exposure Prophylaxis (PrEP), an HIV prevention treatment taken by people at risk of HIV infection, has been in use as a strategy to curtail HIV transmission [2]. Since its launch in 2012, PrEP has been demonstrated to be more effective, with studies showing an estimated reduction in HIV infection between 27% and 99% when compared with no PrEP [2–4]. Pharmacological interventions for PrEP can be taken orally or by injection. These included Apretude, a long-acting injection with antiretroviral cabotegravir given once every two months; Truvada, an oral daily method combining tenofovir, disoproxil, and emtricitabine; as well as Descovy, an oral daily method containing tenofovir alafenamide and emtricitabine [4–7].

Multiple PrEP methods exist with a high prevention rate against HIV infections when used as intended; however, new HIV preventative methods are still required to meet the specific needs of girls and women [8]. This is important, especially in settings where girls and women are at a high risk of sexual violence and in scenarios where they have limited abilities to negotiate for safe sex practices [9]. Dapivirine Vaginal Ring (DVR) was developed to provide girls and women aged 16–45 years more control over HIV prevention [8]. DVR is an approach that promotes women’s sexual rights by providing a discrete HIV preventive technique for women living in disadvantaged situations who may experience sexual violence [9].

Data from phase 3 randomized, double-blind, placebo-controlled trials showed that DVR had a relative risk reduction of HIV infection from 27% to 31% [10,11]. At 12 months, the demand rates for DVR were found to be above 60%, with an acceptance rate of 88.5% [12,13]. While adherence to DVR has been demonstrated to vary between 48% and 90% [14–16], factors associated with adherence (barriers and facilitators) are less clear/have not been summarized across studies [17,18]. Vaginal bleeding may cause some women to remove the DVR due to hygienic concerns, beliefs that the ring could block menstrual flow, fears that the ring would come out with blood or during tampon removal, and fear of overburdening the vagina [3,19]. On the contrary, one study reported that the majority (60%) did not mind wearing the ring during vaginal bleeding and would not remove it (91%) [19].

Strategies to improve ring uptake have been noted to include working with and training healthcare providers with available guides and friendly counseling techniques to encourage usage; working with non-governmental organizations and community health workers to ensure that the ring is provided in a culturally sensitive manner while addressing stigma, side effects, and myths about the rings [10]. There are no pooled estimates of adherence to the DVR [20], limiting the evidence base to guide decision-making and practice [21]. The objective of this systematic review was to summarize the evidence on adherence to DVR for HIV-1 prevention among African women and the associated factors.

## Methods

### Review Registration and Standard Reporting

This review was registered with PROSPERO (registration number CRD42024593018) and also published in a peer reviewed journal [22]. Findings from this study are reported according to the PRISMA guidelines [23].

### Eligibility

We included randomized and quasi-randomized trials, prospective or retrospective cohort studies, case-control studies, longitudinal (one-arm) observational studies (time-series and before-after studies), and case series with more than 10 female participants with data on DVR adherence from Africa aged 16–45 years old. Studies meeting these criteria were included to ensure a comprehensive and relevant response to the research questions. We excluded qualitative studies or studies without final results.

### Information sources

An experienced medical librarian (R.C.) developed search strategies specific to individual databases for the review questions. We searched MEDLINE, Global Health, and EMBASE via OVID platforms and CINAHL via the EBSCO platform from database inception to December 2024 without any language restriction. We also conducted a review of gray literature using Google search as well as the reference lists of all included for possible articles or records of information around DVR. Our search strategy included terms for Dapivirine Vaginal Ring, DVR, and adherence (S1 Appendix).

### Study selection

Pairs of experienced reviewers screened titles and abstracts of identified citations independently, using a standardized, pilot-tested form. Subsequently, reviewers assessed the eligibility of full texts of potentially eligible studies. Reviewers resolved disagreements by discussion or adjudication with a third reviewer (L.M.). We used Covidence online systematic review software to screen titles and abstracts, full-text articles, and to abstract data [24].

### Data abstraction and conversion

Using standardized, pilot-tested forms, pairs of reviewers independently extracted the following data from eligible studies:

(i) study characteristics [author’s name, publication year, study design (observational, quasi-randomized, or randomized), country of origin, and funding source]; (ii) population-related information/factors associated with DVR adherence [median age and age grouping, level of education, marital status, income, partner’s knowledge of ring use, transactional sex, number of episodes of vaginal sex, and use of contraceptive methods such as condoms]; (iii) duration of DVR treatment (all data were converted to months); and (iv) adherence as defined by study authors. In instances where one study had more than one publication, the publication with adherence data was considered for analysis.

Adherence to the ring was typically assessed in two or three categories based on drug release levels: ≤ 0.9 mg (no adherence), > 0.9 mg to 4.0 mg (moderate adherence), and >4.0 mg (good adherence).

For randomized controlled trials, only data from the treatment arm were extracted [11,25]. When adherence was reported in three categories, the highest level was extracted.

In addition, we extracted the total number of follow-up visits and the number of visits where adherence to the DVR was reported. We also recorded the total number of rings distributed and the number of rings that were returned with evidence of good adherence. We also extracted mean and median adherence values, along with their measures of spread (e.g., standard deviations), to estimate mean adherence. Where medians were reported, we converted to means and standard deviations used to estimate standard errors. The 25 mg DVR are designed to release about 4 mg over one month under consistent use. Means closer to 4mg indicate better adherence. To convert means to proportions, we took the mean scores divided by four to get a percentage adherence rate.

As needed, the number of women who were adherent was computed from the percentage adherence rate. Adherence was then standardized into two categories, i.e., Good adherence vs poor adherence, to allow comparisons across studies.

### Risk of bias assessment

The same pairs of reviewers assessed the risk of bias independently. We assessed risk of bias for prevalence of adherence by using the 10-item tool developed by Hoy et al. [26] A judgment of high or low can be assigned to each domain, and an overall judgment of high, low, or moderate was made based on the reviewers’ assessment of all 10 items. All disagreements were resolved through discussion or the involvement of a third reviewer if needed (L.M.). The results of these assessments were plotted as bar charts.

### Statistical analysis

We pooled adherence reported in two or more studies using the DerSimonian–Laird random effects model for proportions and means [27]. For proportions, to stabilize variance, we applied the Freeman–Tukey double arcsine transformation [28]. Heterogeneity was determined by visual inspection of forest plots and I^2^ values. Pool I^2^ estimates were interpreted based on Cochrane categories: 0% to 40%: Might not be important; 30% to 60%: May represent moderate heterogeneity; 50% to 90%: May represent substantial heterogeneity; and 75% to 100%: Considerable heterogeneity [29]. These ranges were also used for making conclusions on the effects of adherence [30]. Study results were described narratively when the number of eligible studies was insufficient for meta-analysis or when substantial conceptual heterogeneity precluded data pooling. Results are presented in forest plots as percentages and 95% confidence intervals or mean and standard deviation. We used Stata (StataCorp, Release 18 BE, College Station, TX, USA) for all statistical analyses.

### Subgroup analysis

There was insufficient data to conduct subgroup analysis.

### Meta-regression analysis

A meta-regression to investigate whether the median age of the participants and the year of study completion was not conducted due to limited observations.

### Assessment of certainty in evidence

We assessed the certainty in our pooled estimates by using the Grading of Recommendations, Assessment, Development, and Evaluation (GRADE) approach [31]. Based on the GRADE five domains of risk of bias, Inconsistency, Indirectness, Imprecision, and Publication Bias, the certainty of evidence was graded as Very Low, Low, Moderate, or High [32].

## Results

### Results of search

**Fig 1:**
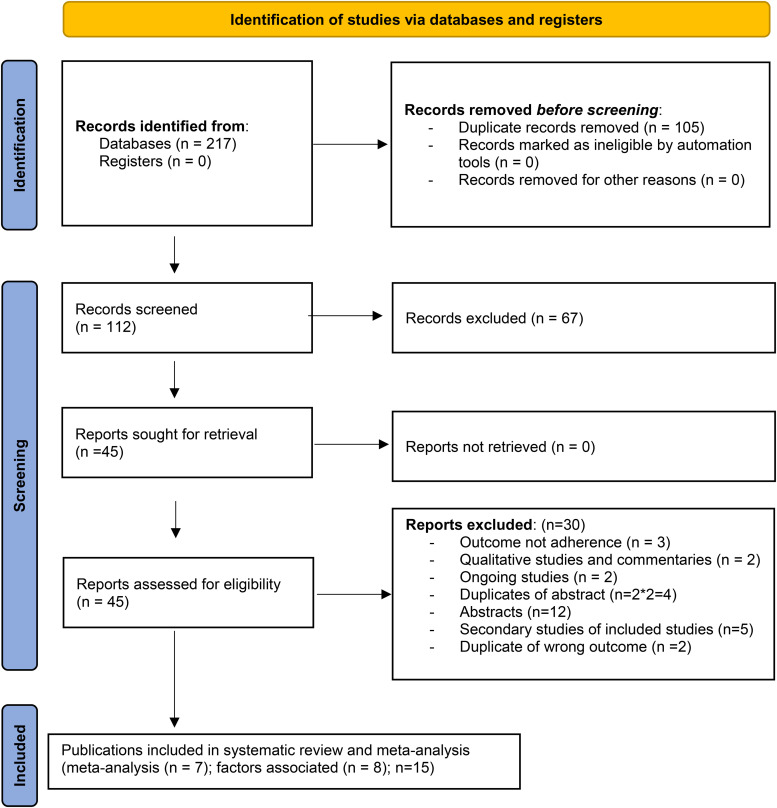
PRISMA flow diagram for study.

Our search retrieved 217 articles (S1 Appendix). After removing duplicates, 112 articles remained, which were then screened based on their titles and abstracts, resulting in 45 articles for full-text screening. Fifteen [14] publications were eligible (seven unique studies) (Fig 1).

### Characteristics of included publications

We found 15 publications [3,10,11,33,20,34–43] of seven unique studies all conducted among women aged 16–45 years in six African countries (Malawi, Tanzania, South Africa, Zimbabwe, Kenya, and Uganda) (Table 1). Across the included studies, adherence to the vaginal ring was measured as proportion with good adherence (4 studies), mean adherence (1 study), number of follow-ups with good adherence (1 study); and 1 study reported both mean adherence and number of returned rings deemed adherent. Eight of the 15 publications reported factors associated with adherence [10,33,34,36–39,42].

**Table 1:** Included publications on Dapivirine Vaginal Ring.

Study ID	Acronym	Treatment/ Control	Country	Sample size	Funding source	Age groups	Year study ended	Design
Palanee-Phillips 2018 [36]	ASPIRE	Dapivirine Vaginal Ring	MWI, UGA, ZWE, and ZAF	2629	Government	18-45	2015	OS
Husnik 2024 [42]	ASPIRE	Dapivirine Vaginal Ring	MWI, UGA, ZWE, and UGA	2629	Government	18-45	2015	OS
Garcia 2021 [43]	ASPIRE	Dapivirine Vaginal Ring	MWI, ZAF, ZWE, and UGA	2629	Government	18-45	2015	OS
Browne 2022 [33]	ASPIRE	Dapivirine Vaginal Ring	ZAF	713	Government	18-45	2015	OS
Roberts 2020 [38]	ASPIRE	Dapivirine Vaginal Ring	MWI, UGA, ZWE, and UGA	2629	Government	18-45	2015	OS
Mayo 2021 [39]	ASPIRE	Dapivirine Vaginal Ring	MWI, UGA, ZWE, and UGA	2629	Government	18-45	2015	OS
Baeten 2016 [10]	ASPIRE	Dapivirine Vaginal Ring	MWI, UGA, ZWE, and UGA	2629	Government	18-45	2015	RCT
Nair 2023 [40]	REACH	Dapivirine ring/daily oral PrEP	UGA, ZWE, and UGA	247	Government	16-21	2021	RCT
Ngure 2024 [37]	REACH	Dapivirine Vaginal Ring/ Oral FTC/TDF	UGA, ZWE, and ZAF	247	Government	16-21	2021	RCT
Stoner 2021 [35]	HOPE	Dapivirine Vaginal Ring	MWI, UGA, ZWE, and UGA	1432	Government	18-45	2018	OS
Baeten 2021 [3]	HOPE	Dapivirine Vaginal Ring	MWI, UGA, ZWE, and UGA	1456	Government	18-45	2018	RCT
Nel 2016a [11]	RING	Dapivirine Vaginal Ring	UGA and ZAF	1959	Private	18-45	2015	RCT
Nel 2021 [20]	DREAM	Dapivirine	UGA and ZAF	941	Government	18-45	2019	RCT
Nel 2016b [34]	No acronym	Dapivirine a/ Placebo Ring	MWI, TZA, KEN, and UGA	280	Private	18-40	2011	RCT
Montgomery 2022 [41]	CHARISMA	Dapivirine Vaginal Ring	ZAF	96	Government	18-45	2017	OS

**MWI**: Malawi, **TZA**: Tanzania, **ZAF**: South Africa, **ZWE**: Zimbabwe, **KEN**: Kenya, **UGA**: Uganda, **OS**: Observational Study, **RCT**: Randomized Controlled Trial.

### Characteristics of included studies

DVR was studied in open-label and phase control trials (I-IV) as a single HIV prevention tool (7 studies) or compared with a placebo ring without Dapivirine (two studies). In two studies, it was compared with other PrEP methods such as Truvada (Table 2). In all studies, adherence was assessed using residual dapivirine levels in returned rings, and only one study used a self-reported measure [34].

**Table 2:** Characteristics of included studies in the meta-analysis.

Primary author & year of publication	Studies acronym name	Study Design	Countries	Sample size	N (m)	Mean (SD)	SE	Total follow-up visits (Adherent visits)	Total rings distributed (Adherent rings)
Baeten 2016 [10]	ASPIRE	RCT	MWI, UGA, ZWE, and UGA	2629	1313 (1103)	NA	NA	NA	NA
Nel 2021 [20]	DREAM	RCT	UGA and ZAF	941	848 (704)	NA	NA	NA	NA
Nair 2023 [40]	REACH	RCT	UGA, ZWE, and UGA	247	123 (70)	NA	NA	NA	NA
Nel 2016b [34]	No acronym	RCT	MWI, TZA, KEN, and UGA	280	140 (101)	NA	NA	NA	NA
Baeten 2021 [3]	HOPE	RCT	MWI, UGA, ZWE, and UGA	1456	NA	3.2 (1.33)	0.04	NA	14,034(12,530)
Montgomery 2022 [41]	CHARISMA	OS	ZAF	96	NA	3.55(1.66)	0.17	NA	NA
Roberts 2020 [38]	ASPIRE	OS	MWI, UGA, ZWE, and UGA	2629	NA	NA	NA	27,904 (20,699)	NA

**MWI**: Malawi, **TZA**: Tanzania, **ZAF**: South Africa, **ZWE**: Zimbabwe, **KEN**: Kenya, **UGA**: Uganda; **N**: Number of people in whom adherence was measured; **m**: Number of people with good adherence, **NA**: Not Applicable, **SD**: Standard Deviation, **SE**: Standard Error, **OS**: Observational Study, **RCT**: Randomized Controlled Trial.

### Characteristics of excluded studies

Among the 30 studies that were excluded from this analysis, three did not report the outcome of interest, three were qualitative and commentary studies, four were duplicates of abstracts, five were secondary studies of included studies, and two did not have the outcomes of interest. Further, 12 were abstracts. Two studies were ongoing [44,45]. A list of excluded studies is reported (S2 Appendix).

### Risk of bias

**Fig 2:**
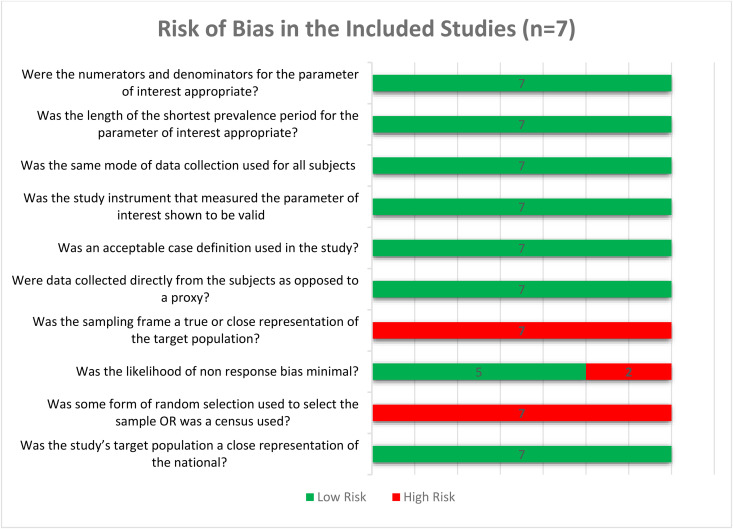
Risk of bias assessment.

The overall risk of bias for most of the studies included was judged to be low. The domains with the most concerns were the non-response bias minimization and the representation of the target population. Four did not minimize the likelihood of non-response bias. None of the seven studies included used random selection. Furthermore, none of the studies used a true sampling frame, and therefore, none met the risk of bias criterion related to sample representativeness. Details of the risk of bias assessments are summarized in Fig 2.

### Mean adherence to Dapivirine Vaginal Ring (DVR)

**Fig 3:**
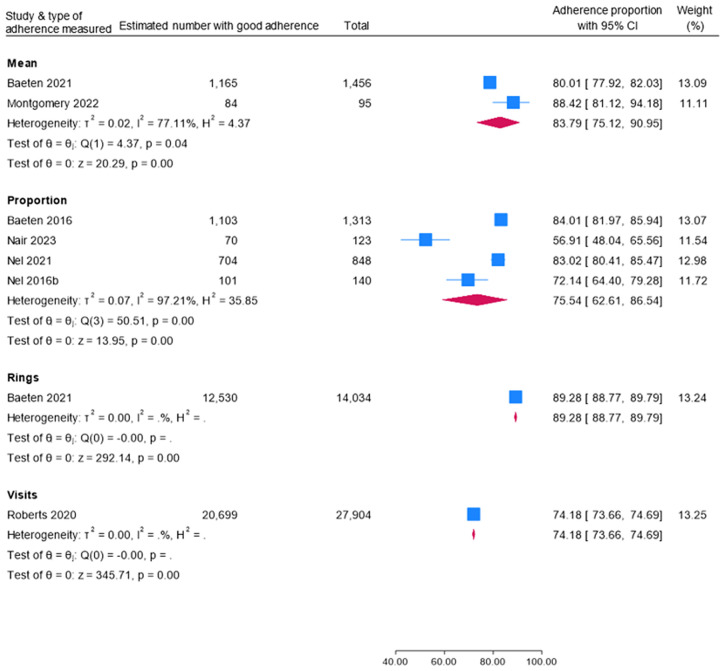
Forest plot of adherence rates to the Dapivirine Vaginal Ring.

The pooled mean adherence to DVR was 83.79% (95% CI 75.12-90.95%); two studies, low certainty of evidence. Heterogeneity was considerable (I^2^ = 77.11%; Fig 3).

### Proportion of women adhering to Dapivirine Vaginal Ring (DVR)

Our meta-analysis revealed a pooled adherence to DVR of 75.54% (95% CI 62.61-86.54%); 4 studies, 2,121 adherent participants; low certainty of evidence. Heterogeneity was considerable (I^2^ = 97.21%; Fig 3).

### Proportions of rings used and follow-up visits reflecting adherence to the Dapivirine Vaginal Ring (DVR)

Overall, 89.28% (95% CI 88.77-89.79%) of rings (1 study) and 74.18% (95% CI 73.66-74.69%) of visits (1 study) showed adherence to the DVR with low certainty of evidence (Fig 3).

### Publication bias

We did not conduct Egger’s test for publication bias, as fewer than 10 studies were included in our meta-analysis.

### GRADE findings

Using the GRADE approach, the certainty of evidence was assessed across seven studies included in the meta-analysis. Our findings demonstrated very low certainty of evidence, suggesting limited confidence in the estimated effect. Certainty of evidence was rated down due to risk of bias and imprecision owing to high risk of bias in the sampling frame, closeness to the target population, random selection, and a deficiency in sample size. We also downgraded the certainty of evidence by two levels for inconsistency, due to a high degree of heterogeneity across the forest plots, with I² values ranging from 75% to 99%. Additionally, we downgraded for indirectness due to differences between the adherence measures used in the included studies (e.g., adherence categorization) (Table 3).

**Table 3:** Summary of findings table for certainty of evidence.

Outcomes	Pooled estimates (95% CI)	№ of participants/visits/rings (studies)	Certainty of the evidence (GRADE)
Proportions	83.79% (95% CI 75.12-90.95%)	2,424 (4)	⨁◯◯◯ Very low ^a, b, c, d^
Mean adherence	75.54% (95% CI 62.61-86.54%)	1,552 (2)	⨁◯◯◯ Very low^a, b, c, d^
Visits	74.18% (95% CI 73.66-74.69%)	27,904 (1)	⨁◯◯◯ Very low^a,e^
Returned rings	89.28% (95% CI 88.77-89.79%)	14,034 (1)	⨁◯◯◯ Very low^a,e^

**Explanations**

^a^Most studies were rated as having an overall high risk of bias.

^b^I^2^ > 70%; point estimates and corresponding 95% Confidence Interval (CI) not overlapping.

^c^Self-reported adherence measured heterogeneously; some dichotomize it into two levels (good/poor) while others used a three-level categorization (poor/moderate/good). Rated down once for indirectness.

^d^Downgraded one due to wide 95% CI and Optimal Information Size (OIS) not met.

^e^Downgraded twice due to wide 95% CI and OIS not met.

### Factors associated with Dapivirine Vaginal Ring (DVR) adherence

A total of 8 studies reported factors associated with DVR adherence. These factors are categorized as user, partner, ring, and environmental characteristics, and further described as facilitators or barriers to DVR use. At the user level, factors such as older age, higher education, and income facilitated improved adherence whereas unstable living conditions were linked to reduced use. Partner-related factors, including disclosure of ring use, facilitated adherence while partner discomfort (e.g., feeling the ring during sexual intercourse) acted as a barrier. At the product level, difficulty with insertion and sensation of the ring during sex were identified as barriers. Environmental factors, such as supportive social norms, were found to improve adherence (Table 4) [10].

**Table 4:** Factors associated with DVR adherence in 8 studies.

User characteristics	Partner characteristics	Ring characteristics	Environmental characteristics
**Facilitators** • Age/age group [10,33,37] • Education [37] • Country [43] • Income [37] • Vaginal sex, three months before enrolment [40] • Transactional sex [37] • Use of contraceptive methods [10,37] • Participant engagement activities [43] **Barriers** • Unstable house [37]	**Facilitators** • Partners’ (primary) knowledge of ring use/ had visited the clinic [38] • Disclosure of product use [37] **Barriers** • Partner felt ring during sex [39]	**Facilitators** • None **Barriers** • Felt ring during sex [39] • Very/somewhat difficult to insert [39] • Sometimes/usually uncomfortable to have ring inside every day [39] • One or more grade two or higher adverse events related to DVR during crossover periods [40] **•**Influence of other PrEP methods [40] • Some/most of the time aware of the ring during normal activities [39]	**Facilitators** • Social contributors with highly influential views [35] • Supportive social opinions about the ring [35] • Social opinions shared by participants [35]

## Discussion

Overall, despite very low certainty of evidence, adherence to the DVR among women appeared to be good, with a pooled mean adherence estimate of 84% (95% CI: 75%-91%), though the individual mean study estimates varied from 56-84%. Overall, adherence among women was high, with a pooled estimate of 76% (95% CI: 63%-87%). Individual studies consistently reported higher mean estimates (80%-88%), suggesting generally good adherence across settings. Similarly, adherence based on the number of rings used and visits attended was good, estimated at 74% (95% CI 74–75%) and 89% (95% CI 89–90%), respectively. Adherence to the DVR was influenced by multiple factors, including individual characteristics (e.g., education level and country), partner-related factors (e.g., whether the partner felt the ring during sex), ring-related factors (e.g., difficulty inserting the ring), and environmental factors (e.g., discussing the study with non-study staff)

The good adherence observed in this study might be explained by the diverse strategies used to engage community members, which is observed in the number of follow-ups. Further, women included in the studies might have had a better understanding of the ring and are keen on health matters, even though findings were heterogeneous. The heterogeneity in DVR adherence observed across included studies may be explained by differences in study populations, settings, and methodologies. Similarly, in the factors associated, we identified that the age group of women was associated with adherence to DVR [10,33,37]. Specifically, younger women under the age of 26 are known to have lower adherence rates than their older counterparts [10]. This may mean they need support to adhere to and improve treatment outcomes. Low adherence in this age group has not been reported only in the use of DVR but also in research focusing on sexual health interventions, where young people may fail to adhere to alternative PrEP techniques [46–48]. Factors influencing adherence to DVR use include side effects such as urinary tract infection, vaginal discharge, vulvovaginal pruritus (itching), vulvovaginitis, and pelvic pain. These adverse effects may negatively influence adherence. It is important to note that DVR use does not protect against sexually transmitted infections; therefore, additional preventive measures, such as condom use, are recommended [10,37]. In contrast, some studies have shown that younger women are likely to have higher adherence to similar tools if they perceive that they are at high risk of HIV infection [49]. Therefore, implementing well-tailored personalized support, providing education about the perceived benefits of PrEP to this population, facilitating access to trusted healthcare providers, and accommodating lengthy trials to create room for empowerment on the tool could improve adherence within this age group [50].

On the other hand, older age was found to be associated with being adherent to the DVR, irrespective of the existing known factors that limit adherence among young women. This comparatively good adherence may exist due to women’s prior knowledge of related products such as the NuvaRing, and the female condom [51,52], as well as the independence they have over their health matters. Also, some studies have found that despite good adherence among older women, some still purposefully remove the ring [53]. Factors that lead to such removal include discomfort during use/sex, concerns of harm, a doctor’s request, not on a salary-based income, rumors about sickness and infertility [54,55]. Evidence from another continent suggests that three out of four of women at menopause had perfect adherence to the ring [56]. In this scenario, women preferred the ring because it was easy to use and did not interfere with erection [56]. Thus, one might conclude a better adherence to the ring in this age group as compared to others, which needs, though not yet, to be studied in Africa.

### Strengths and limitations

The strengths of this systematic review and meta-analysis lie in the breadth of the search, the novelty of the research question, the analytical methods, and the narrative synthesis of factors associated with DVR adherence. Most limitations of our review originate from the underlying evidence. These include limited data availability, leading to the inability to conduct subgroup analyses and preventing pooling of all studies conducted in Africa to date. The risk of bias was low for most studies. There was a serious heterogeneity. This could be attributed to differences in study designs, dosing in mother studies or trials, and other clinical characteristics. We employed a random effects model that incorporates heterogeneity, however, we suggest caution when interpreting these results.

### Future directions based on study findings

Given the findings of this study, there is a need to advance implementation research to determine circumstances that would help users adhere to the ring above the current rates. This could mean resizing the ring to limit the chance of partners feeling the ring during sexual intercourse and advancing the ring’s life span to limit it being user-dependent. Even though some findings already exist on young women [40], these are not sufficient to capture specifics on adherence among young women in Africa. Given the already known low adherence within this population, as well as the differences in risky health behaviors [57] that may exist across Africa [58], it is essential to investigate uptake and adherence within this population in a diversified context. Furthermore, there is a need to consider strategies to subsidize costs related to the use of the ring, especially for young people whose income levels often affect their use and adherence to sexual and reproductive health services and tools such as DVR [59,60]. Additionally, a standardized method to capture adherence should be employed for all studies being carried out. This would help in the effective evaluation of findings and a better understanding of implementation strategies. Furthermore, there is a need for study adherence across different categories of women, including those at menopause. By capturing a wide and similar population across the globe, there would be room for a comprehensive comparative evaluation of DVR adherence worldwide, providing guidelines for implementation. The unavailable of data and inability to use some of the already published findings in their current format require advanced methods such as Individual Patient Meta-analysis to help better explain factors associated with DVR and reasons behind the considerable level of heterogeneity between studies.

## Conclusion

Our study is the most comprehensive review of the level of DVR adherence and associated factors. In agreement with most studies, DVR adherence is lower in younger individuals. The overall adherence to DVR is suggested to be good, though with very low certainty of evidence. Approaches that promote adherence while limiting side effects, such as sexually transmitted infections, and implementation research targeting women with different characteristics, including those at menopause, are needed. Particularly, Individual Patient Data Meta-analysis will provide better estimates of DVR adherence. These findings suggest that DVR should not be used as a sole tool for HIV prevention.

## Supporting information

S1 Appendix Database search. (DOCX)

S2 Appendix Characteristics of excluded studies [61–81]. (DOCX)

S1 Checklist PRISMA checklist. From: Page MJ, McKenzie JE, Bossuyt PM, Boutron I, Hoffmann TC, Mulrow CD, et al. The PRISMA 2020 statement: an updated guideline for reporting systematic reviews. BMJ 2021;372:n71. doi: 10.1136/bmj.n71. This work is licensed under CC BY 4.0. To view a copy of this license, visit https://creativecommons.org/licenses/by/4.0/.
(DOCX)
