# Supplementary material for: Estimated adherence to the Dapivirine Vaginal Ring and its associated factors among African women: A systematic review and meta-analysis
Source: PLOS Glob Public Health. 2026 May 18;6(5):e0006422. doi: 10.1371/journal.pgph.0006422 (PMC13183192; doi:10.1371/journal.pgph.0006422)
Supplement: S1 Appendix — (DOCX) [file pgph.0006422.s001.docx]

**Data search**

**Summary of search and strategy dapivirine**

| MEDLINE | 45 |
| --- | --- |
| Embase | 98 |
| CINAHL | 17 |
| Global Health | 17 |
| Google Scholar | 40 |
| Subtotal | 217 |
| -dupes | -105 |
| Total | 112 |

1. Used Africa filter by Pienaar, E., Grobler, L., Busgeeth, K., Eisinga, A. and Siegfried, N. (2011), Developing a geographic search filter to identify randomised controlled trials in Africa: finding the optimal balance between sensitivity and precision. Health Information & Libraries Journal, 28: 210-215. https://doi.org/10.1111/j.1471-1842.2011.00936.x
2. **September 10, 2024**
3. **MEDLINE (OVID)**
4. Database: OVID Medline Epub Ahead of Print, In-Process & Other Non-Indexed Citations, Ovid MEDLINE(R) Daily and Ovid MEDLINE(R) 1946 to Present
5. Search Strategy:
6. --------------------------------------------------------------------------------
7. 1 dapivirine.mp. (304)
8. 2 (aids 105293 or aids105293 or r 147681 or r147681 or tmc 120 or tmc120).mp. (33)
9. 3 (DPVVR or DPV-VR).mp. [mp=title, book title, abstract, original title, name of substance word, subject heading word, floating sub-heading word, keyword heading word, organism supplementary concept word, protocol supplementary concept word, rare disease supplementary concept word, unique identifier, synonyms, population supplementary concept word, anatomy supplementary concept word] (3)
10. 4 or/1-3 (322)
11. 5 (therapy or regimen or intervention or PrEP or prophyla*).mp. [mp=title, book title, abstract, original title, name of substance word, subject heading word, floating sub-heading word, keyword heading word, organism supplementary concept word, protocol supplementary concept word, rare disease supplementary concept word, unique identifier, synonyms, population supplementary concept word, anatomy supplementary concept word] (7032056)
12. 6 4 and 5 (193)
13. 7 (adheren* or complian*).mp. [mp=title, book title, abstract, original title, name of substance word, subject heading word, floating sub-heading word, keyword heading word, organism supplementary concept word, protocol supplementary concept word, rare disease supplementary concept word, unique identifier, synonyms, population supplementary concept word, anatomy supplementary concept word] (435275)
14. 8 6 and 7 (74)
15. 9 Africa.mp. or exp Africa/ (408587)
16. 10 (Africa* or Algeria or Angola or Benin or Botswana or Burkina Faso or Burundi or Cameroon or Canary Islands or Cape Verde or Central African Republic or Chad).mp. [mp=title, book title, abstract, original title, name of substance word, subject heading word, floating sub-heading word, keyword heading word, organism supplementary concept word, protocol supplementary concept word, rare disease supplementary concept word, unique identifier, synonyms, population supplementary concept word, anatomy supplementary concept word] (409064)
17. 11 (Comoros or Congo or Democratic Republic of Congo or Djibouti or Egypt or Equatorial Guinea or Eritrea or Ethiopia or Gabon or Gambia or Ghana or Guinea or Guinea Bissau or Ivory Coast or Cote d'Ivoire or Jamahiriya or Jamahiryia or Kenya or Lesotho or Liberia or Libya or Libia).mp. [mp=title, book title, abstract, original title, name of substance word, subject heading word, floating sub-heading word, keyword heading word, organism supplementary concept word, protocol supplementary concept word, rare disease supplementary concept word, unique identifier, synonyms, population supplementary concept word, anatomy supplementary concept word] (310725)
18. 12 (Madagascar or Malawi or Mali or Mauritania or Mauritius or Mayote or Morocco or Mozambique or Mocambique or Namibia or Niger or Nigeria or Principe or Reunion or Rwanda or Sao Tome or Senegal or Seychelles or Sierra Leone or Somalia or South Africa or St Helena or Sudan or Swaziland).mp. [mp=title, book title, abstract, original title, name of substance word, subject heading word, floating sub-heading word, keyword heading word, organism supplementary concept word, protocol supplementary concept word, rare disease supplementary concept word, unique identifier, synonyms, population supplementary concept word, anatomy supplementary concept word] (207502)
19. 13 (Tanzania or Togo or Tunisia or Uganda or Western Sahara or Zaire or Zambia or Zimbabwe or Central Africa or Central African or West Africa or West African or Western Africa or Western African or East Africa or East African or Eastern Africa or Eastern African or North Africa or North African or Northern Africa or Northern African or South African or Southern Africa or Southern African or subSaharan Africa or subSaharan African).mp. [mp=title, book title, abstract, original title, name of substance word, subject heading word, floating sub-heading word, keyword heading word, organism supplementary concept word, protocol supplementary concept word, rare disease supplementary concept word, unique identifier, synonyms, population supplementary concept word, anatomy supplementary concept word] (139410)
20. 14 or/9-13 (829957)
21. 15 ("guinea pig" or "guinea pigs" or "aspergillus niger").tw. (107613)
22. 16 14 not 15 (722344)
23. 17 8 and 16 (45)
24. **Database: Embase <1974 to 2024 September 09>**
25. Search Strategy:
26. --------------------------------------------------------------------------------
27. 1 dapivirine.mp. or dapivirine/ (923)
28. 2 (aids 105293 or aids105293 or r 147681 or r147681 or tmc 120 or tmc120).mp. [mp=title, abstract, heading word, drug trade name, original title, device manufacturer, drug manufacturer, device trade name, keyword heading word, floating subheading word, candidate term word] (207)
29. 3 (DPVVR or DPV-VR).mp. [mp=title, abstract, heading word, drug trade name, original title, device manufacturer, drug manufacturer, device trade name, keyword heading word, floating subheading word, candidate term word] (7)
30. 4 or/1-3 (933)
31. 5 (therapy or regimen or intervention or PrEP or prophyla*).mp. [mp=title, abstract, heading word, drug trade name, original title, device manufacturer, drug manufacturer, device trade name, keyword heading word, floating subheading word, candidate term word] (10981217)
32. 6 4 and 5 (663)
33. 7 (adheren* or complian*).mp. [mp=title, abstract, heading word, drug trade name, original title, device manufacturer, drug manufacturer, device trade name, keyword heading word, floating subheading word, candidate term word] (713214)
34. 8 6 and 7 (246)
35. 9 Africa.mp. or exp Africa/ (486815)
36. 10 (Africa* or Algeria or Angola or Benin or Botswana or Burkina Faso or Burundi or Cameroon or Canary Islands or Cape Verde or Central African Republic or Chad).mp. [mp=title, abstract, heading word, drug trade name, original title, device manufacturer, drug manufacturer, device trade name, keyword heading word, floating subheading word, candidate term word] (495543)
37. 11 (Comoros or Congo or Democratic Republic of Congo or Djibouti or Egypt or Equatorial Guinea or Eritrea or Ethiopia or Gabon or Gambia or Ghana or Guinea or Guinea Bissau or Ivory Coast or Cote d'Ivoire or Jamahiriya or Jamahiryia or Kenya or Lesotho or Liberia or Libya or Libia).mp. [mp=title, abstract, heading word, drug trade name, original title, device manufacturer, drug manufacturer, device trade name, keyword heading word, floating subheading word, candidate term word] (306300)
38. 12 (Madagascar or Malawi or Mali or Mauritania or Mauritius or Mayote or Morocco or Mozambique or Mocambique or Namibia or Niger or Nigeria or Principe or Reunion or Rwanda or Sao Tome or Senegal or Seychelles or Sierra Leone or Somalia or South Africa or St Helena or Sudan or Swaziland).mp. [mp=title, abstract, heading word, drug trade name, original title, device manufacturer, drug manufacturer, device trade name, keyword heading word, floating subheading word, candidate term word] (244424)
39. 13 (Tanzania or Togo or Tunisia or Uganda or Western Sahara or Zaire or Zambia or Zimbabwe or Central Africa or Central African or West Africa or West African or Western Africa or Western African or East Africa or East African or Eastern Africa or Eastern African or North Africa or North African or Northern Africa or Northern African or South African or Southern Africa or Southern African or subSaharan Africa or subSaharan African).mp. [mp=title, abstract, heading word, drug trade name, original title, device manufacturer, drug manufacturer, device trade name, keyword heading word, floating subheading word, candidate term word] (158444)
40. 14 or/9-13 (935577)
41. 15 ("guinea pig" or "guinea pigs" or "aspergillus niger").tw. (106803)
42. 16 14 not 15 (828774)
43. 17 8 and 16 (98)
44. **CINAHL (EBSCO)**

| Tue, September 10, 2024 2:09:23 PM |
| --- |

| **#** | **Query** | **Limiters/Expanders** | **Last Run Via** | **Results** |
| --- | --- | --- | --- | --- |
| S14 | S8 AND S13 | Search modes - Proximity | Interface - EBSCOhost Research Databases Search Screen - Advanced Search Database - CINAHL | 17 |
| S13 | S11 not S12 | Search modes - Proximity | Interface - EBSCOhost Research Databases Search Screen - Advanced Search Database - CINAHL | 251,289 |
| S12 | TX ("guinea pig" or "guinea pigs" or "aspergillus niger") | Search modes - Proximity | Interface - EBSCOhost Research Databases Search Screen - Advanced Search Database - CINAHL | 2,935 |
| S11 | S9 OR S10 | Search modes - Proximity | Interface - EBSCOhost Research Databases Search Screen - Advanced Search Database - CINAHL | 254,224 |
| S10 | TX ( Africa* or Algeria or Angola or Benin or Botswana or Burkina Faso or Burundi or Cameroon or Canary Islands or Cape Verde or Central African Republic or Chad ) OR TX ( Comoros or Congo or Democratic Republic of Congo or Djibouti or Egypt or Equatorial Guinea or Eritrea or Ethiopia or Gabon or Gambia or Ghana or Guinea or Guinea Bissau or Ivory Coast or Cote d'Ivoire or Jamahiriya or Jamahiryia or Kenya or Lesotho or Liberia or Libya or Libia ) OR TX ( Madagascar or Malawi or Mali or Mauritania or Mauritius or Mayote or Morocco or Mozambique or Mocambique or Namibia or Niger or Nigeria or Principe or Reunion or Rwanda or Sao Tome or Senegal or Seychelles or Sierra Leone or Somalia or South Africa or St Helena or Sudan or Swaziland ) OR TX ( Tanzania or Togo or Tunisia or Uganda or Western Sahara or Zaire or Zambia or Zimbabwe or Central Africa or Central African or West Africa or West African or Western Africa or Western African or East Africa or East African or Eastern Africa or Eastern African or North Africa or North African or Northern Africa or Northern African or South African or Southern Africa or Southern African or subSaharan Africa or subSaharan African ) | Search modes - Proximity | Interface - EBSCOhost Research Databases Search Screen - Advanced Search Database - CINAHL | 254,224 |
| S9 | (MH "Africa+") OR "Africa" | Search modes - Proximity | Interface - EBSCOhost Research Databases Search Screen - Advanced Search Database - CINAHL | 113,990 |
| S8 | S6 AND S7 | Search modes - Proximity | Interface - EBSCOhost Research Databases Search Screen - Advanced Search Database - CINAHL | 19 |
| S7 | TX (adheren* or complian*) | Search modes - Proximity | Interface - EBSCOhost Research Databases Search Screen - Advanced Search Database - CINAHL | 170,250 |
| S6 | S4 AND S5 | Search modes - Proximity | Interface - EBSCOhost Research Databases Search Screen - Advanced Search Database - CINAHL | 34 |
| S5 | TX (therapy or regimen or intervention or PrEP or prophyla*) | Search modes - Proximity | Interface - EBSCOhost Research Databases Search Screen - Advanced Search Database - CINAHL | 2,440,393 |
| S4 | S1 OR S2 OR S3 | Search modes - Proximity | Interface - EBSCOhost Research Databases Search Screen - Advanced Search Database - CINAHL | 79 |
| S3 | TX (DPVVR or DPV-VR) | Search modes - Proximity | Interface - EBSCOhost Research Databases Search Screen - Advanced Search Database - CINAHL | 1 |
| S2 | TX (aids 105293 or aids105293 or r 147681 or r147681 or tmc 120 or tmc120) | Search modes - Proximity | Interface - EBSCOhost Research Databases Search Screen - Advanced Search Database - CINAHL | 3 |
| S1 | "dapivirine" | Search modes - Proximity | Interface - EBSCOhost Research Databases Search Screen - Advanced Search Database - CINAHL | 77 |

1. **Global Health (OVID)**
2. **Database: Global Health <1973 to 2024 Week 36>**
3. Search Strategy:
4. --------------------------------------------------------------------------------
5. 1 dapivirine.mp. [mp=abstract, title, original title, broad terms, heading words, cabicodes words] (128)
6. 2 (aids 105293 or aids105293 or r 147681 or r147681 or tmc 120 or tmc120).mp. [mp=abstract, title, original title, broad terms, heading words, cabicodes words] (20)
7. 3 (DPVVR or DPV-VR).mp. [mp=abstract, title, original title, broad terms, heading words, cabicodes words] (3)
8. 4 or/1-3 (141)
9. 5 (therapy or regimen or intervention or PrEP or prophyla*).mp. [mp=abstract, title, original title, broad terms, heading words, cabicodes words] (750036)
10. 6 4 and 5 (74)
11. 7 (adheren* or complian*).mp. [mp=abstract, title, original title, broad terms, heading words, cabicodes words] (77878)
12. 8 6 and 7 (34)
13. 9 Africa.mp. or exp Africa/ (303458)
14. 10 (Africa* or Algeria or Angola or Benin or Botswana or Burkina Faso or Burundi or Cameroon or Canary Islands or Cape Verde or Central African Republic or Chad).mp. [mp=abstract, title, original title, broad terms, heading words, cabicodes words] (340738)
15. 11 (Comoros or Congo or Democratic Republic of Congo or Djibouti or Egypt or Equatorial Guinea or Eritrea or Ethiopia or Gabon or Gambia or Ghana or Guinea or Guinea Bissau or Ivory Coast or Cote d'Ivoire or Jamahiriya or Jamahiryia or Kenya or Lesotho or Liberia or Libya or Libia).mp. [mp=abstract, title, original title, broad terms, heading words, cabicodes words] (115659)
16. 12 (Madagascar or Malawi or Mali or Mauritania or Mauritius or Mayote or Morocco or Mozambique or Mocambique or Namibia or Niger or Nigeria or Principe or Reunion or Rwanda or Sao Tome or Senegal or Seychelles or Sierra Leone or Somalia or South Africa or St Helena or Sudan or Swaziland).mp. [mp=abstract, title, original title, broad terms, heading words, cabicodes words] (137499)
17. 13 (Tanzania or Togo or Tunisia or Uganda or Western Sahara or Zaire or Zambia or Zimbabwe or Central Africa or Central African or West Africa or West African or Western Africa or Western African or East Africa or East African or Eastern Africa or Eastern African or North Africa or North African or Northern Africa or Northern African or South African or Southern Africa or Southern African or subSaharan Africa or subSaharan African).mp. [mp=abstract, title, original title, broad terms, heading words, cabicodes words] (281861)
18. 14 or/9-13 (374416)
19. 15 ("guinea pig" or "guinea pigs" or "aspergillus niger").tw. (19612)
20. 16 14 not 15 (354804)
21. 17 8 and 16 (17)
22. **GoogleScholar**
23. allintitle: Dapivirine adherence OR compliance
24. Yields 40
