# Supplementary material for: Estimated adherence to the Dapivirine Vaginal Ring and its associated factors among African women: A systematic review and meta-analysis
Source: PLOS Glob Public Health. 2026 May 18;6(5):e0006422. doi: 10.1371/journal.pgph.0006422 (PMC13183192; doi:10.1371/journal.pgph.0006422)
Supplement: S2 Appendix — (DOCX) [file pgph.0006422.s002.docx]

**Characteristics of excluded studies**

| **Characteristics** | **Studies** |
| --- | --- |
| Ongoing studies | (43,44) |
| Outcome not adherence | (16,60,61) |
| Qualitative and commentary studies | (54,62) |
| Duplicates of the abstract | (63,64) |
| Abstracts | (61,65–75) |
| Secondary studies of the included studies | (18,76–79) |
| Duplicate of wrong outcome | (80) |
